# Human skin dendritic cells in health and disease

**DOI:** 10.1016/j.jdermsci.2014.08.012

**Published:** 2015-02

**Authors:** Muzlifah Haniffa, Merry Gunawan, Laura Jardine

**Affiliations:** a Institute of Cellular Medicine, Newcastle University, NE2 4HH, UK; b Department of Dermatology, Newcastle Upon Tyne NHS Trust, NE1 4LP, UK

**Keywords:** Dendritic cells, Mononuclear phagocytes, Antigen presenting cells, Skin

## Abstract

Dendritic cells (DCs) are specialized antigen presenting cells abundant in peripheral tissues such as skin where they function as immune sentinels. Skin DCs migrate to draining lymph node where they interact with naïve T cells to induce immune responses to microorganisms, vaccines, tumours and self-antigens. In this review, we present the key historical developments and recent advances in human skin DC research. We also integrate the current understanding on the origin and functional specializations of DC subsets in healthy skin with findings in inflammatory skin diseases focusing on psoriasis and atopic eczema. A comprehensive understanding of the dynamic changes in DC subsets in health and disease will form a strong foundation to facilitate the clinical translation of DC-based therapeutic and vaccination strategies.

## Highlights

• Human skin dendritic cells (DCs) are heterogenous and functionally specialised.
• Factor XIIIa+ dermal dendrocytes are resident dermal macrophages.
• Dermal CD14+ cells, previously defined as DCs, are monocyte-derived macrophages.
• Dynamic changes occur in the composition of recruited ‘inflammatory’ DCs and resident DCs in inflamed skin.

## 1 Introduction

Dendritic cells (DCs) are a heterogeneous population of leukocytes that are critical in orchestrating immune responses. In humans, the logistical difficulties of studying tissue DCs have led to the extensive use of peripheral blood *in vitro* monocyte-derived DCs (mo-DCs) as an experimental tool. The *in vivo* equivalent of mo-DCs may be DCs seen in inflammation rather than healthy tissue. The skin is an accessible epithelial barrier rich in antigen presenting cells (APCs) and has been used as a model tissue to study primary DCs in humans. In this review, we will outline the current understanding of the composition, function and origin of human skin DCs in health and two common inflammatory skin diseases, psoriasis and atopic eczema.

## 2 Skin dendritic cells

The demonstration of MHC Class II, Fc and C3 receptors on epidermal Langerhans cells (LCs) 109 years after their initial discovery by Paul Langerhans in 1868, confirmed their identity as immune cells and promoted the use of human skin as a convenient source to study tissue DCs [1], [2], [3]. These initial studies on murine and human LCs formed the paradigm for ‘migratory’ tissue DCs which sample antigen in their local microenvironment and migrate to draining lymph node where they interact with T lymphocytes to initiate a specific immune response [4]. The first interrogation of DCs in the human dermis was undertaken by immunostaining for Factor XIIIa (FXIIIa) which identified branching spindle shaped cells called ‘dermal dendrocytes’ [5]. This was followed by the observation in 1993 that dermal myeloid DCs, distinct from epidermal LCs, spontaneously migrated from skin explants cultured *ex vivo*. Analysis of migrated cells identified two dermal DC subsets characterized by the expression of CD1a and CD14 [6], [7]. However, *in situ* analysis of the human dermis revealed CD1c^+^ DCs which co-express CD1a and FXIIIa^+^CD14^+^CD163^+^ dermal macrophages [8]. The puzzling observation of two myeloid DCs within cells migrating spontaneously from skin explants but only one subset identifiable *in situ* was explained by the overlapping antigen profile of CD14^+^ DCs with dermal macrophages. There are several features that distinguish CD14^+^ DCs from macrophages: (1) morphology: macrophages contain dense cytoplasmic melanin granules, (2) flow cytometry: macrophages have high scatter properties which result in autofluorescence easily identifiable in the FITC channel (excitation/emission: 488/530(20)), (3) migratory behavior: only dermal CD14^+^ DCs migrate spontaneously from skin explants cultured *ex viv*o, (4) adherence: macrophages are adherent to tissue culture plastic and (5) turnover kinetics: macrophages are reconstituted at a significantly slower rate by donor-derived cells following hematopoietic stem cell (HSC) transplantation [9].

In addition to CD1c^+^ DCs and CD14^+^ DCs, CD141^hi^ DCs were recently identified in skin and other peripheral tissues [10]. Although high expression of CD141 characterize this subset, this antigen is also expressed by all CD14^+^ DCs and a subset of CD1c^+^ DCs [11]. An important distinction of CD141^hi^ DCs from the other DC subsets is the lack of CD14 expression and lower expression of CD11c [10]. In the dermis, myeloid DCs are located more superficially than macrophages, which are present deeper and primarily perivascular in distribution [12]. Whether the three myeloid DC subsets occupy distinct microanatomical spaces is unknown. Gene expression studies suggest that human skin CD141^hi^ DCs are homologous to murine CD103^+^/CD8^+^ DCs and CD1c^+^ DCs are homologous to CD11b^+^CD24^+^CD64^−^ DCs (reviewed in [13]). Our recent analysis showed that dermal CD14^+^ ‘DCs’ are monocyte-derived cells, which are transcriptionally similar to FXIIIa^+^ macrophages [14]. In contrast to myeloid DCs, plasmacytoid DCs (pDCs) are virtually undetectable in healthy skin but are recruited during inflammation [8], [15], [16]. pDCs are located in lymphoid tissues such as lymph node and tonsil [17], [18]. In addition to pDCs and tissue ‘migratory’ myeloid DCs, draining lymph node also contains ‘resident’ myeloid DCs. Lymph node ‘resident’ CD1c^+^ and CD141^+^ DCs are HLADR^lo^ and CD11c^hi^, distinguishable from HLADR^hi^CD11c^lo^ ‘migratory’ DCs [10].

What is the biological need for different DC subsets? It is important that division into DC subsets is not simply a trivial classification exercise. A considerable body of evidence has accumulated over the years demonstrating specialized immune functions for the various DC subsets. These studies have used migrated primary cells or from enzymatically-digested skin and in vitro CD34^+^ hematopoietic stem cell (HSC)-derived CD14^+^ DCs and CD1a^+^ LCs [19], [20], [21], [22], [23], [24]. A summary of the different functions described for skin DC subsets can be found in Table 1.

A further consideration is the phenotypic stability of skin DCs. DC subsets identified from enzymatic-digestion and spontaneous migration have been shown to have similar antigenic profile. Although this suggests phenotypic stability, altered proportion of DC subsets upon *ex vivo* cytokine treatments has been documented suggesting cellular plasticity [22], [25], [26]. Whether plasticity within differentiated resident populations is an important feature *in vivo* is uncertain. The demonstration of long-lived recipient-derived macrophages after allogeneic HSC transplant, despite the rapid repopulation of dermal DCs by donor-derived cells, suggests that dermal macrophages do not differentiate into resident skin DCs [9].

## 3 Origin of human skin dendritic cells

DCs arise from a bone marrow HSC-derived lineage dependent on the receptor tyrosine kinase FLT3 [27], [28], [29] (Fig. 2). Patients deficient in blood monocytes and DCs due to IRF8 and GATA2 mutation lack dermal DC subsets, have reduced numbers of macrophages but intact LCs [30], [31]. This implies that dermal DCs are directly dependant on circulating monocytes and/or DCs or a shared HSC-derived precursor. In contrast, macrophages and LCs are likely to arise from alternative precursors *e.g.* embryonic or tissue-resident precursors, or are simply long-lived and turnover very slowly. In mice, LCs were shown to arise from embryonic progenitors which seed the skin prior to birth [32], [33]. It is possible that similar embryonic precursors directly contribute to human LCs. Both human and murine LCs also possess local proliferative potential [34], [35].

**Fig. 1:**
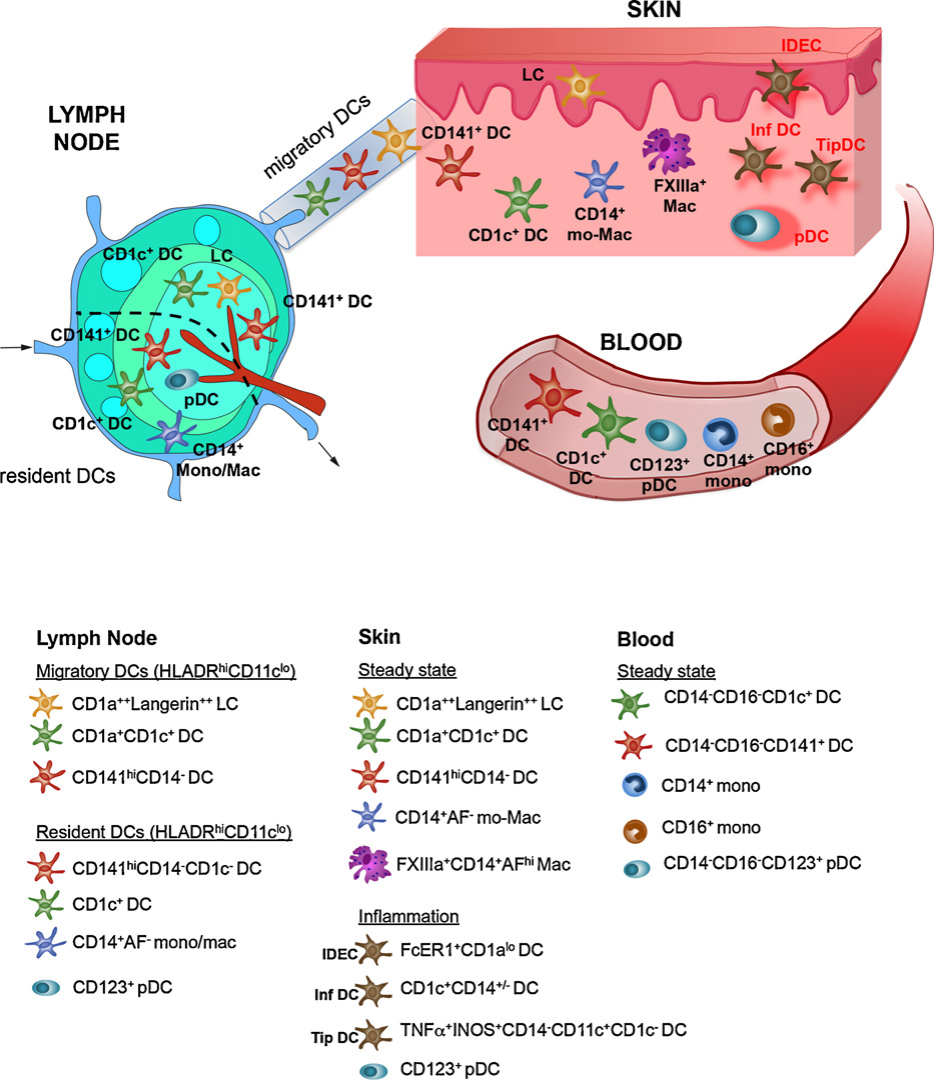
Distribution of human dendritic cells, monocytes and macrophages in skin, blood and lymph nodes. Changes during inflammation are indicated in red text. pDC = plasmacytoid DCs, Mac = macrophage, mono = monocytes, mo-Mac = monocyte-derived macrophage, inf DC = inflamatory DCs, IDEC = inflammatory dendritic epidermal cell, TipDC = TNFa and iNOS producing DC.

The specific contributions of circulating blood DCs and monocytes to skin DC subsets are still unclear. Human blood DCs were identified in 1982 as cells expressing MHC Class II, negative for lineage markers defining T, B and NK cells (CD3, CD19, CD20 and CD56) with potent allostimulatory properties [36], [37]. The Lin^−^ClassII^+^ blood compartment contains human monocytes and DC subsets, which all except pDCs, express the integrin CD11c. Human monocyte subsets can be identified by the expression of CD14 and CD16. DCs are found within the CD14^−^CD16^−^ fraction and can be characterized by the expression of CD1c and CD141/BDCA3 [38]. The phenotypic differences between DCs initially identified in peripheral tissues (CD1c^+^ and CD14^+^ ‘DCs’) and blood (CD1c^+^ and CD141^+^ DCs) was an obstacle to establish their precise relationships easily. As skin CD14^+^ ‘DCs’ also express CD141, which is further upregulated during spontaneous migration from skin explant culture, it was initially thought to be the equivalent of blood CD141^+^ DCs [11]. The identification of tissue CD14^−^CD141^hi^ DCs, distinct from CD14^+^ ‘DCs’ and CD1c^+^ DCs, corresponding to blood CD141^+^ DCs, has facilitated the alignment of DC networks in peripheral blood and skin as shown in Fig. 1. A proportion of cells within peripheral blood CD16^+^ monocyte population expressing 6-Sulfo LacNAc (SLAN), called SLAN DCs, have also been described [39]. In healthy skin, SLAN^+^ cells have been found but unlike other DCs, do not express CD11c [40].

**Fig. 2:**
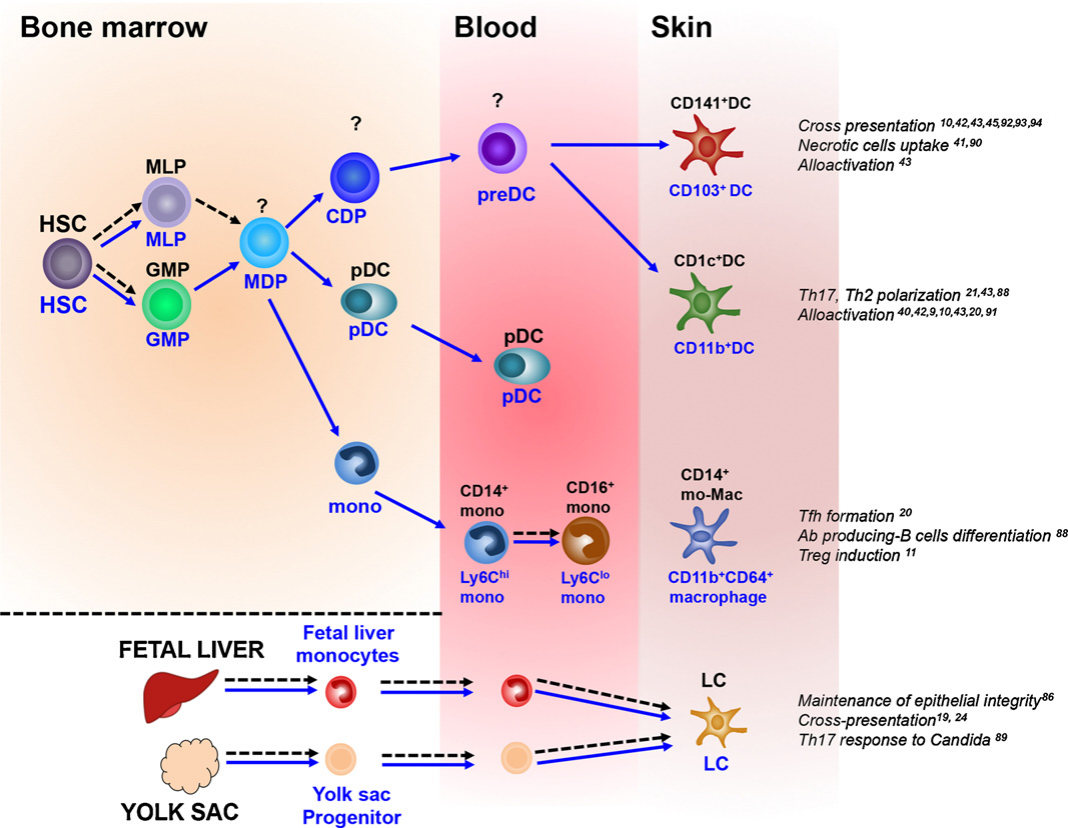
Ontogeny of human and mouse dendritic cells in the steady state. Precursors, monocytes and DC subsets are annotated in black for human, and the mouse homologs are annotated in blue underneath the cell type. Question marks indicate unknown identity for the relevant species. Proven origin and relationships are indicated by solid arrows and speculative relationships by dashed arrows. Functional properties of each dendritic cell subset are specified adjacent each cell type. HSC = hematopoietic stem cells, MLP = mixed lymphoid progenitors, GMP = granulocyte macrophage progenitors, MDP = monocyte–macrophage/DC precursor, CDP = common DC precursor, preDC = precursor of myeloid DC, pDC = plasmacytoid DCs, mac = macrophage, mono = monocytes and mo-Mac = monocyte-derived macrophage [88], [89], [90], [91], [92], [93], [94].

The human and mice DC networks appears to be conserved (Fig. 2) [10], [41], [42], [43], [44], [45], [46]. Inter-species homology predicts that the human CD141^+^ DCs in blood and skin arise from a precursor that precludes a monocyte stage. Blood CD141^+^ DCs upregulate CD1c and CD1a upon co-culture with skin and express the the skin homing receptor CLA suggesting that blood CD141^+^ DCs may be the immediate precursors of skin CD141^hi^ DCs [10]. Human CD141^+^ and CD1c^+^ DCs possess a unique phenotype transcription signature distinct from monocytes and macrophages. The murine homologs of dermal CD14^+^ cells are dermal CD11b^+^CD64^+^ macrophages (Fig. 2).

## 4 Skin dendritic cells in inflammation and disease

The function of DCs as cutaneous sentinels and instigators of T cell responses suggests a key role for these cells in inflammatory skin diseases. We are beginning to understand the contribution of DC to the pathogenesis of psoriasis and atopic dermatitis (AD). An important consideration in studying DCs in inflamed skin is to distinguish resident DCs that are normally present in skin from cells recruited during inflammation. This is difficult for a number of reasons: (i) there are no unique markers to identify recruited cells and (ii) resident subsets may have an altered phenotype in inflammatory environment. Furthermore, ‘snapshot’ analysis of inflamed skin does not take into account the dynamic state of migratory DCs which affects the nature and quantity of skin DCs at a given time point during disease evolution. Functional differences of DC subsets in inflammation may also be skewed by the tissue microenvironment. In this section, we will review the contribution of DCs in psoriasis and AD pathogenesis with reference to these difficulties.

### 4.1 Dendritic cell phenotype in inflamed skin

Animal models suggest that inflammation is accompanied by monocyte-derived DC accumulation in tissues. Mice infected with *Listeria monocytogenes* accumulate DCs in spleen. These DCs produce TNFα and iNOS and are called TipDCs [47]. Inflammatory DCs have also been described in a murine cutaneous Leishmania model of skin inflammation [48]. In both models, infiltrating cells express murine DC markers (CD11c, MHC II, CD80, CD86 and DEC205) alongside monocyte (CD11b, Ly6C) and macrophage-associated antigens (Mac-3, F4/80).

A recent study on inflamed human synovial and ascitic fluid [49], compartments where few resident cells are present in healthy state, revealed inflammatory DCs which expressed HLA-DR, CD11c and CD1c. These cells also express varying levels of CD1a, CD14, CD206, FcER1 and SIRPα. It is difficult to translate this finding into skin where CD11c, HLA-DR and CD1c expression would also identify resident dermal CD1c DCs. In psoriasis, DCs have been recognized as a significant proportion of inflammatory lesions [50]. Chemerin production by dermal fibroblasts, endothelial and mast cells in psoriasis lesional and peri-lesional skin attracts pDC in the initial stage of plaque formation [16]. The downstream upregulation of Type I IFN genes results in subsequent myeloid inflammatory DC recruitment [51], [52]. Dermal CD11c^+^ cells in psoriasis skin outnumber lymphocytes and coincide with areas of TNFα and iNOS production [50]. The majority of CD11c^+^ cells express high levels of HLA-DR as well as CD40 and CD86 [50]. By immunohistochemistry, many CD11c^+^ cells are positive for SLAN [53]. The absence of CD14, CD1c, CD1a and langerin distinguishes these inflammatory DC from resident subsets. Co-localized detection of TNFα and iNOS has lead to the suggestion that these cells equate to TipDCs seen in murine models. Cells expressing CD14 but lower levels of HLA-DR, in keeping with monocytes, are a small proportion of inflammatory lesions [50].

In AD skin, CD1a^+^CD11b^+^CD1c^+^ myeloid DCs and pDCs [51], [54], [55], [56] have been observed. Both subsets express the high affinity IgE receptor, FcER1 [56]. Myeloid DCs isolated from AD epidermal suspensions are called inflammatory dendritic epidermal cells (IDEC) [57]. IDECs are distinct from resident Langerhans cells by their lower expression of CD1a and lack of Birbeck granules, but it is not clear how IDECs relate to dermal resident CD1c^+^ DC, which can co-express CD1a and CD206.

### 4.2 Origins of inflammatory dendritic cells and their homeostasis in inflammation

Although inflammatory skin lesions contain increased numbers of DC, the precise origin of recruited DCs remains unclear. In animal models, inflammatory DCs derive from the Ly6C^hi^ monocytes [47], [48], [58], [59], [60], which are equivalent to CD14 human monocytes [61]. This differentiation is dependent on MyD88, a key regulator of inflammatory cytokine signaling [62]. While GMCSF is used *in vitro* to model inflammatory DC, its presence *in vivo* is not essential for monocyte-derived inflammatory DC differentiation [63]. Demonstrating cell ontogeny is more challenging in humans, but transcriptomic analysis showed that inflammatory CD11c^+^HLA-DR^+^CD16^−^CD1c^+^ DCs from synovial and ascitic fluids resemble in vitro mo-DCs [49]. However, convergent genetic reprogramming can occur in both conventional and mo-DC subsets upon microbial stimuli [64]. SLAN^+^TipDCs found in psoriasis have been suggested to originate from blood SLAN^+^ DCs [53]. This conclusion is based on patterns of chemokine receptors, cytokine production and margination of SLAN^+^ cells along dermal capillaries. Analysis of cytokine production in a moDC model of IDECs supports their derivation from CD14 monocytes [65]. The contribution of blood or skin CD1c^+^ DCs, which express FcER1, as precursors of IDECs has not been evaluated. While it is clear that recruited cells are important for the generation of inflammatory DCs, it is difficult to ascertain the precise contribution, if any, of *in situ* resident DC differentiation to this pool.

During the influx of monocytes to inflamed tissue, steady state mechanisms of DC homeostasis are stressed and may lead to alterations in resident population origin. An excellent example is the differential precursor requirement for LCs in steady state and inflammation. LCs are seeded from embryonic precursors during foetal development and proliferate in quiescent skin to self-renew [32], [34]. However, during inflammation, LC may arise from monocytes or bone marrow precursors and have an accelerated turnover, as demonstrated by more rapid transition to donor-derived LC in cutaneous graft *versus* host disease following bone marrow transplantation [66], [67], [68]. In mice, tissue infiltration with monocytes promotes monopoiesis at the expense of other myeloid differentiation [69]. Skewing of myeloid development pathways has not been demonstrated in humans, but may have significant effects in chronic inflammation. Insufficient replacement of resident DCs could contribute to loss of tolerance and secondary infection.

### 4.3 Functional properties of dendritic cells in inflammation

Inflammatory DCs contribute to beneficial immune responses in murine infectious models. The TipDCs in murine *L. monocytogenes* infection model have allostimulatory capacity in mixed leucocyte reactions but are not required for effective CD4 and CD8 T cell priming *in vivo.* Their beneficial role in clearing bacteria is attributed to TNF and iNOS production [47]. However, inflammatory DC in murine cutaneous Leishmaniasis do prime naïve T cells and contribute to pathogen-clearing Th1 responses *in vivo* [48]. Protective CD8, and Th2 responses have been demonstrated in influenza, vaccination and sensitization models respectively [59], [70], [71]. Inflammatory DC may also shape adaptive immunity *in situ* by activating tissue-resident effector memory T cells [72].

Current understanding of psoriasis reveals multiple contributions by DCs in disease pathogenesis. IFNα produced by pDCs during initial plaque formation [52] leads to IL-23 and IL-17 upregulation in the skin. IL-23 polarizes Th17 cell differentiation and also potentiates IL-17 production by a variety of immune cells such as neutrophils, mast cells and γδ T cells in psoriasis lesion [73]. The genetic association with the IL-23/Th17 pathway and the efficacy of anti-IL-23 and anti-IL-17 therapies support the importance of IL-23 and IL-17 in psoriasis pathogenesis [50], [74]. Recent reports show that anti-TNFα therapy may also target IL-23 and IL-17 pathway in clearing psoriasis [75]. DCs in normal and psoriasis skin are capable of producing IL-23 [76]. In addition, SLAN^+^TipDCs found in psoriasis skin have been shown to prime naïve T cells to produce Th1/17 cytokines [77] similar to DCs from inflammatory fluids [49]. The expression of Th1 and Th17 recruiting chemokines CXCL1, CXCL8 and CCL20 is upregulated in psoriasis skin but the precise contribution of DCs to chemokine secretion in psoriasis is unknown [78]. Interestingly, the observation that peri-lesional psoriasis skin spontaneously develops into psoriasis plaque following engraftment onto mice suggests that skin resident leukocytes alone are sufficient for disease manifestation [79]. How skin DCs directly modulate resident T cells in lesional skin warrants further exploration.

Disruption of skin barrier function due to filaggrin deficiency is an important predisposing factor for AD [80]. The barrier-breakdown signal, TLSP, is produced by keratinocytes and is critical in the pathogenesis of AD (reviewed in [81]). TSLP-activated DCs have been shown to be potent stimulators of naïve T cells and drive Th2 cytokine production. LCs which are in the appropriate anatomical compartment have been shown to be TSLP responsive [82]. Th2 producing lymphocytes are recruited to tissue *via* CCL17, CCL18 and CCL22 signaling [78]. These chemokines have been detected in myeloid DCs and LCs in AD skin [78]. Th22 cytokines also feature in AD lesions, and their production can be stimulated by pDCs [83], [84]. Crosslinking IgE is important in later stages of AD pathogenesis and its high affinity receptor, FcERI, is expressed by LCs, IDECs and pDCs [55], [56]. FcERI-activation of both *in vitro*-derived LCs and IDECs yields a proinflammatory response [85].

Although it is clear that the skin APC compartment expands in psoriasis and AD, the subsequent fate of APCs during disease progression and inflammation resolution is unknown. There is little evidence that inflammatory DCs in psoriasis and AD migrate into lymphatics or re-enter blood circulation. Alternative possibilities include cell death in the skin or differentiation into a resident subset.

## 5 Conclusion

The human skin has a rich network of DCs which are heterogeneous and functionally specialized. Recent progress in distinguishing DC subsets from resident macrophages and the characterization of the dynamic populations in inflammatory states has begun to shed light on their role in skin homeostasis and pathology. An enhanced understanding of skin DCs origin, homeostasis, function and pathogenic role in disease will provide novel avenues to be exploited for clinical therapy.

## Funding

We acknowledge funding from The Wellcome Trust, UK (WT088555; M.H. and WT097941; LJ); British Skin Foundation (M.H. and M.G.); and AXA Research Fund (M.G.).

## Figures and Tables

**Table 1:** Functional studies on skin DC subsets.

**Reference**	**Subset**	**Isolation and generation**			**Function**						
**Migrate**	**Digest**	***In vitro***	**Cytokine**	**Alloactivation**	**Th2 polarization**	**Th1 polarization**	**Cross-presentation**	**Cross-priming**	**Memory/recall response**
Caux et al. [19]	CD1a+ (CD1a+CD14-)			•		+++					
CD14+ (CD1a-CD14+)			•		+++					

Klechevsky et al. [20]	LCs (CD1ahi CD14-HLA-DR+CD207+)	•			IL-15, IL-8	+++	+++	++			
CD14+ dDCs (CD1a-CD14+HLA-DR+)	•			IL-10, IL-6, MCP-1, IL-12p40, IL-1β, GM-CSF, TNFα	+	+	++			
CD1a+ dDCs (CD1adim CD14-HLA-DR+)	•			IL-15, IL-8	++	++	++			
CD1a+ CD14- LCs			•		+++	+++	++	+++	+++	++
CD1a-CD14+ DCs			•		+	++	++	+	+	+++

Morelli et al. [21]	CD1a+CD14- LCs	•			IL-10, TGFβ1	+++	++	+++			+
CD1a-CD14- DDCs	•			IL-10, TGFβ1	++	++	+++			+
CD1a-CD14+ preLCs	•			IL-10, TGFβ1	+	+	++			

Angel et al. [23]	dLCs (CD1ahi CD207+ CD14-)		•								
CD1a+ dDCs (CD1adim CD207- CD14-)		•			+++					
CD14+ dDCs (CD1a-CD207- CD14+)		•			++					

Haniffa et al. [9]	HLA-DR+CD14-CD1a+ dDCs		•		IL-1, IL-6, IL-23, IL-10	+++					+
HLA-DR+CD14+CD1a- dDCs		•								+
HLA-DR+CD14+CD1a-FXIIIa+ dMACs		•		IL-1, IL-6	+					

Haniffa et al. [10]	CD141 DCs (CD141hiCD11clo-intCD1clo)		•		TNFα, CXCL10				++++		
CD1c DCs (CD141loCD11chiCD1c+)		•		TNFα, IL-10, IL-8				+++		
CD14+ DCs		•		IL-1β, IL-6, IL-8, IL-10				+		
LCs		•		CXCL10, IL-8				+++		

Matthews et al. [86]	CD14+CD1a- migDCs	•			IL-6, IL-10, TNFα, IL-1β	+	+++	+			+
CD1a+CD14- migDCs	•			IL-6	+++	+	+++			+
CD14-CD1a- migDCs	•									
LCs		•			-					
CD1a+ dDCs		•			-					

Polak et al. [24]	LCs	•							+++		
CD11c+ dDC	•							+		

Penel-Sotirakis et al. [87]	LCs	•			TNFα, IL-6	+++					++
CD1c+CD14- dDCs	•			TNFα, IL-6	++					++
CD14+ dDCs	•			TNFα	+					+

Fujita et al. [78]	LCs (HLA-DR+CD207+)		•			+++	++	++			
CD1c+ dDC (HLA-DRhiCD11c+1c+)		•			++	+	+			

## References

[1] Stingl G., Wolff-Schreiner E.C., Pichler W.J., Gschnait F., Knapp W., Wolff K. (1977). Epidermal Langerhans cells bear Fc and C3 receptors. Nature.

[2] Rowden G., Lewis M.G., Sullivan A.K. (1977). Ia antigen expression on human epidermal Langerhans cells. Nature.

[3] Klareskog L., Tjernlund U., Forsum U., Peterson P.A. (1977). Epidermal Langerhans cells express Ia antigens. Nature.

[4] Schuler G., Steinman R.M. (1985). Murine epidermal Langerhans cells mature into potent immunostimulatory dendritic cells in vitro. J. Exp. Med..

[5] Cerio R., Griffiths C.E., Cooper K.D., Nickoloff B.J., Headington J.T. (1989). Characterization of factor XIIIa positive dermal dendritic cells in normal and inflamed skin. Br. J. Dermatol..

[6] Lenz A., Heine M., Schuler G., Romani N. (1993). Human and murine dermis contain dendritic cells. Isolation by means of a novel method and phenotypical and functional characterization. J Clin Investig.

[7] Nestle F.O., Zheng X.G., Thompson C.B., Turka L.A., Nickoloff B.J. (1993). Characterization of dermal dendritic cells obtained from normal human skin reveals phenotypic and functionally distinctive subsets. J. Immunol..

[8] Zaba L.C., Fuentes-Duculan J., Steinman R.M., Krueger J.G., Lowes M.A. (2007). Normal human dermis contains distinct populations of CD11c+BDCA-1+ dendritic cells and CD163+FXIIIA+ macrophages. J Clin Investig.

[9] Haniffa M., Ginhoux F., Wang X.N., Bigley V., Abel M., Dimmick I. (2009). Differential rates of replacement of human dermal dendritic cells and macrophages during hematopoietic stem cell transplantation. J. Exp. Med..

[10] Haniffa M., Shin A., Bigley V., McGovern N., Teo P., See P. (2012). Human tissues contain CD141(hi) cross-presenting dendritic cells with functional homology to mouse CD103(+) nonlymphoid dendritic cells. Immunity.

[11] Chu C.-C., Ali N., Karagiannis P., Di Meglio P., Skowera A., Napolitano L. (2012). Resident CD141 (BDCA3)^+^ dendritic cells in human skin produce IL-10 and induce regulatory T cells that suppress skin inflammation. J Exp Med.

[12] Wang X.N., McGovern N., Gunawan M., Richardson C., Windebank M., Siah T.W. (2014). A three-dimensional atlas of human dermal leukocytes, lymphatics, and blood vessels. J Investig Dermatol.

[13] Haniffa M., Collin M., Ginhoux F. (2013). Ontogeny and functional specialization of dendritic cells in human and mouse. Adv. Immunol..

[14] McGovern N., Schlitzer A., Gunawan M., Jardine L., Shin A., Poyner E. (2004). Human Dermal CD14(+) Cells are a Transient Population of Monocyte-Derived Macrophages. Immunity.

[15] Nestle F.O., Conrad C., Tun-Kyi A., Homey B., Gombert M., Boyman O. (2005). Plasmacytoid predendritic cells initiate psoriasis through interferon-alpha production. J.Exp.Med.

[16] Albanesi C., Scarponi C., Pallotta S., Daniele R., Bosisio D., Madonna S. (2009). Chemerin expression marks early psoriatic skin lesions and correlates with plasmacytoid dendritic cell recruitment. J. Exp. Med..

[17] Grouard G., Rissoan M.C., Filgueira L., Durand I., Banchereau J., Liu Y.J. (1997). The enigmatic plasmacytoid T cells develop into dendritic cells with interleukin (IL)-3 and CD40-ligand. J. Exp. Med..

[18] Cox K., North M., Burke M., Singhal H., Renton S., Aqel N. (2005). Plasmacytoid dendritic cells (PDC) are the major DC subset innately producing cytokines in human lymph nodes. J. Leukoc. Biol..

[19] Caux C., Vanbervliet B., Massacrier C., Dezutter-Dambuyant C., de Saint-Vis B., Jacquet C. (1996). CD34+ hematopoietic progenitors from human cord blood differentiate along two independent dendritic cell pathways in response to GM-CSF+ TNF alpha. J. Exp. Med..

[20] Klechevsky E., Morita R., Liu M., Cao Y., Coquery S., Thompson-Snipes L. (2008). Functional specializations of human epidermal langerhans cells and CD14+ dermal dendritic cells. Immunity.

[21] Morelli A.E., Rubin J.P., Erdos G., Tkacheva O.A., Mathers A.R., Zahorchak A.F. (2005). Larregina AT: CD4+ T cell responses elicited by different subsets of human skin migratory dendritic cells. J. Immunol..

[22] de Gruijl T.D., Sombroek C.C., Lougheed S.M., Oosterhoff D., Buter J., van den Eertwegh A.J. (2006). A postmigrational switch among skin-derived dendritic cells to a macrophage-like phenotype is predetermined by the intracutaneous cytokine balance. J. Immunol..

[23] Angel C.E., George E., Brooks A.E., Ostrovsky L.L., Brown T.L., Dunbar P.R. (2006). Cutting edge: CD1a+ antigen-presenting cells in human dermis respond rapidly to CCR7 ligands. J. Immunol..

[24] Polak M.E., Newell L., Taraban V.Y., Pickard C., Healy E., Friedmann P.S. (2012). CD70–CD27 interaction augments CD8+ T-cell activation by human epidermal Langerhans cells. J Investig Dermatol.

[25] Lindenberg J.J., Oosterhoff D., Sombroek C.C., Lougheed S.M., Hooijberg E., Stam A.G. (2013). IL-10 conditioning of human skin affects the distribution of migratory dendritic cell subsets and functional T cell differentiation. PLoS One.

[26] Larregina A.T., Morelli A.E., Spencer L.A., Logar A.J., Watkins S.C., Thomson A.W. (2001). Dermal-resident CD14+ cells differentiate into Langerhans cells. Nat. Immunol..

[27] Maraskovsky E., Daro E., Roux E., Teepe M., Maliszewski C.R., Hoek J. (2000). In vivo generation of human dendritic cell subsets by Flt3 ligand. Blood.

[28] McKenna H.J., Stocking K.L., Miller R.E., Brasel K., De Smedt T., Maraskovsky E. (2000). Mice lacking flt3 ligand have deficient hematopoiesis affecting hematopoietic progenitor cells, dendritic cells, and natural killer cells. Blood.

[29] Chen W., Chan A.S., Dawson A.J., Liang X., Blazar B.R., Miller J.S. (2005). FLT3 ligand administration after hematopoietic cell transplantation increases circulating dendritic cell precursors that can be activated by CpG oligodeoxynucleotides to enhance T-cell and natural killer cell function. Biol. Blood Marrow Transplant..

[30] Hambleton S., Salem S., Bustamante J., Bigley V., Boisson-Dupuis S., Azevedo J. (2011). IRF8 mutations and human dendritic-cell immunodeficiency. N. Engl. J. Med..

[31] Bigley V., Haniffa M., Doulatov S., Wang X.N., Dickinson R., McGovern N. (2011). The human syndrome of dendritic cell, monocyte, B and NK lymphoid deficiency. J. Exp. Med..

[32] Hoeffel G., Wang Y., Greter M., See P., Teo P., Malleret B. (2012). Adult Langerhans cells derive predominantly from embryonic fetal liver monocytes with a minor contribution of yolk sac-derived macrophages. J. Exp. Med..

[33] Schulz C., Gomez Perdiguero E., Chorro L., Szabo-Rogers H., Cagnard N., Kierdorf K. (2012). A lineage of myeloid cells independent of Myb and hematopoietic stem cells. Science.

[34] Merad M., Manz M.G., Karsunky H., Wagers A., Peters W., Charo I. (2002). Langerhans cells renew in the skin throughout life under steady-state conditions. Nat. Immunol..

[35] Chorro L., Sarde A., Li M., Woollard K.J., Chambon P., Malissen B. (2009). Langerhans cell (LC) proliferation mediates neonatal development, homeostasis, and inflammation-associated expansion of the epidermal LC network. J. Exp. Med..

[36] Van Voorhis W.C., Hair L.S., Steinman R.M., Kaplan G. (1982). Human dendritic cells. Enrichment and characterization from peripheral blood. J. Exp. Med..

[37] O’Doherty U., Peng M., Gezelter S., Swiggard W.J., Betjes M., Bhardwaj N. (1994). Human blood contains two subsets of dendritic cells, one immunologically mature and the other immature. Immunology.

[38] MacDonald K.P., Munster D.J., Clark G.J., Dzionek A., Schmitz J., Hart D.N. (2002). Characterization of human blood dendritic cell subsets. Blood.

[39] Schakel K., von Kietzell M., Hansel A., Ebling A., Schulze L., Haase M. (2006). Human 6-sulfo LacNAc-expressing dendritic cells are principal producers of early interleukin-12 and are controlled by erythrocytes. Immunity.

[40] Gunther C., Starke J., Zimmermann N., Schakel K. (2012). Human 6-sulfo LacNAc (slan) dendritic cells are a major population of dermal dendritic cells in steady state and inflammation. Clin. Exp. Dermatol..

[41] Robbins S.H., Walzer T., Dembele D., Thibault C., Defays A., Bessou G. (2008). Novel insights into the relationships between dendritic cell subsets in human and mouse revealed by genome-wide expression profiling. Genome Biol..

[42] Bachem A., Guttler S., Hartung E., Ebstein F., Schaefer M., Tannert A. (2010). Superior antigen cross-presentation and XCR1 expression define human CD11c+CD141+ cells as homologues of mouse CD8+ dendritic cells. J. Exp. Med..

[43] Crozat K., Guiton R., Contreras V., Feuillet V., Dutertre C.A., Ventre E. (2010). The XC chemokine receptor 1 is a conserved selective marker of mammalian cells homologous to mouse CD8alpha+ dendritic cells. J. Exp. Med..

[44] Jongbloed S.L., Kassianos A.J., McDonald K.J., Clark G.J., Ju X., Angel C.E. (2010). Human CD141+ (BDCA-3)+ dendritic cells (DCs) represent a unique myeloid DC subset that cross-presents necrotic cell antigens. J. Exp. Med..

[45] Poulin L.F., Salio M., Griessinger E., Anjos-Afonso F., Craciun L., Chen J.L. (2010). Characterization of human DNGR-1+ BDCA3+ leukocytes as putative equivalents of mouse CD8alpha+ dendritic cells. J. Exp. Med..

[46] Schlitzer A., McGovern N., Teo P., Zelante T., Atarashi K., Low D. (2013). IRF4 transcription factor-dependent CD11b+ dendritic cells in human and mouse control mucosal IL-17 cytokine responses. Immunity.

[47] Serbina N.V., Salazar-Mather T.P., Biron C.A., Kuziel W.A., Pamer E.G. (2003). TNF/iNOS-producing dendritic cells mediate innate immune defense against bacterial infection. Immunity.

[48] Leon B., Lopez-Bravo M., Ardavin C. (2007). Monocyte-derived dendritic cells formed at the infection site control the induction of protective T helper 1 responses against Leishmania. Immunity.

[49] Segura E., Touzot M., Bohineust A., Cappuccio A., Chiocchia G., Hosmalin A. (2013). Human inflammatory dendritic cells induce th17 cell differentiation. Immunity.

[50] Lowes M.A., Chamian F., Abello M.V., Fuentes-Duculan J., Lin S.L., Nussbaum R. (2005). Increase in TNF-alpha and inducible nitric oxide synthase-expressing dendritic cells in psoriasis and reduction with efalizumab (anti-CD11a). Proc. Natl. Acad. Sci. U. S. A..

[51] Wollenberg A., Mommaas M., Oppel T., Schottdorf E.M., Gunther S., Moderer M. (2002). Expression and function of the mannose receptor CD206 on epidermal dendritic cells in inflammatory skin diseases. J Investig Dermatol.

[52] Nestle F.O., Conrad C., Tun-Kyi A., Homey B., Gombert M., Boyman O. (2005). Plasmacytoid predendritic cells initiate psoriasis through interferon-alpha production. J. Exp. Med..

[53] Hansel A., Gunther C., Ingwersen J., Starke J., Schmitz M., Bachmann M. (2011). Human slan (6-sulfo LacNAc) dendritic cells are inflammatory dermal dendritic cells in psoriasis and drive strong TH17/TH1 T-cell responses. J. Allergy Clin. Immunol..

[54] Wollenberg A., Wagner M., Gunther S., Towarowski A., Tuma E., Moderer M. (2002). Plasmacytoid dendritic cells: a new cutaneous dendritic cell subset with distinct role in inflammatory skin diseases. J Investig Dermatol.

[55] Bieber T., Kraft S., Geiger E., Wollenberg A., Koch S., Novak N. (2000). Fc [correction of Ec] epsilon RI expressing dendritic cells: the missing link in the pathophysiology of atopic dermatitis?. J. Dermatol..

[56] Stary G., Bangert C., Stingl G., Kopp T. (2005). Dendritic cells in atopic dermatitis: expression of FcepsilonRI on two distinct inflammation-associated subsets. Int. Arch. Allergy Immunol..

[57] Wollenberg A., Kraft S., Hanau D., Bieber T. (1996). Immunomorphological and ultrastructural characterization of Langerhans cells and a novel, inflammatory dendritic epidermal cell (IDEC) population in lesional skin of atopic eczema. J Investig Dermatol.

[58] Cheong C., Matos I., Choi J.H., Dandamudi D.B., Shrestha E., Longhi M.P. (2010). Microbial stimulation fully differentiates monocytes to DC-SIGN/CD209(+) dendritic cells for immune T cell areas. Cell.

[59] Plantinga M., Guilliams M., Vanheerswynghels M., Deswarte K., Branco-Madeira F., Toussaint W. (2013). Conventional and monocyte-derived CD11b(+) dendritic cells initiate and maintain T helper 2 cell-mediated immunity to house dust mite allergen. Immunity.

[60] Tamoutounour S., Guilliams M., Montanana Sanchis F., Liu H., Terhorst D., Malosse C. (2013). Origins and functional specialization of macrophages and of conventional and monocyte-derived dendritic cells in mouse skin. Immunity.

[61] Ziegler-Heitbrock L., Ancuta P., Crowe S., Dalod M., Grau V., Hart D.N. (2010). Nomenclature of monocytes and dendritic cells in blood. Blood.

[62] Serbina N.V., Kuziel W., Flavell R., Akira S., Rollins B., Pamer E.G. (2003). Sequential MyD88-independent and -dependent activation of innate immune responses to intracellular bacterial infection. Immunity.

[63] Greter M., Helft J., Chow A., Hashimoto D., Mortha A., Agudo-Cantero J. (2012). GM-CSF controls nonlymphoid tissue dendritic cell homeostasis but is dispensable for the differentiation of inflammatory dendritic cells. Immunity.

[64] Manh T.P., Alexandre Y., Baranek T., Crozat K., Dalod M. (2013). Plasmacytoid, conventional, and monocyte-derived dendritic cells undergo a profound and convergent genetic reprogramming during their maturation. Eur. J. Immunol..

[65] Novak N., Kraft S., Haberstok J., Geiger E., Allam P., Bieber T. (2002). A reducing microenvironment leads to the generation of FcepsilonRIhigh inflammatory dendritic epidermal cells (IDEC). J. Invest. Dermatol..

[66] Ginhoux F., Tacke F., Angeli V., Bogunovic M., Loubeau M., Dai X.M. (2006). Langerhans cells arise from monocytes in vivo. Nat. Immunol..

[67] Sere K., Baek J.H., Ober-Blobaum J., Muller-Newen G., Tacke F., Yokota Y. (2012). Two distinct types of Langerhans cells populate the skin during steady state and inflammation. Immunity.

[68] Collin M.P., Hart D.N., Jackson G.H., Cook G., Cavet J., Mackinnon S. (2006). The fate of human Langerhans cells in hematopoietic stem cell transplantation. J. Exp. Med..

[69] Serbina N.V., Hohl T.M., Cherny M., Pamer E.G. (2009). Selective expansion of the monocytic lineage directed by bacterial infection. J. Immunol..

[70] Ballesteros-Tato A., Leon B., Lund F.E., Randall T.D. (2010). Temporal changes in dendritic cell subsets, cross-priming and costimulation via CD70 control CD8(+) T cell responses to influenza. Nat. Immunol..

[71] Kool M., Soullie T., van Nimwegen M., Willart M.A., Muskens F., Jung S. (2008). Alum adjuvant boosts adaptive immunity by inducing uric acid and activating inflammatory dendritic cells. J. Exp. Med..

[72] Wakim L.M., Waithman J., van Rooijen N., Heath W.R., Carbone F.R. (2008). Dendritic cell-induced memory T cell activation in nonlymphoid tissues. Science.

[73] Acosta-Rodriguez E.V., Rivino L., Geginat J., Jarrossay D., Gattorno M., Lanzavecchia A. (2007). Surface phenotype and antigenic specificity of human interleukin 17-producing T helper memory cells. Nat. Immunol..

[74] Nair R.P., Duffin K.C., Helms C., Ding J., Stuart P.E., Goldgar D. (2009). Genome-wide scan reveals association of psoriasis with IL-23 and NF-kappaB pathways. Nat. Genet..

[75] Zaba L.C., Cardinale I., Gilleaudeau P., Sullivan-Whalen M., Suarez Farinas M., Fuentes-Duculan J. (2007). Amelioration of epidermal hyperplasia by TNF inhibition is associated with reduced Th17 responses. J. Exp. Med..

[76] Lee E., Trepicchio W.L., Oestreicher J.L., Pittman D., Wang F., Chamian F. (2004). Increased expression of interleukin 23 p19 and p40 in lesional skin of patients with psoriasis vulgaris. J. Exp. Med..

[77] Cragg M.S., Walshe C.A., Ivanov A.O., Glennie M.J. (2005). The biology of CD20 and its potential as a target for mAb therapy. Curr. Dir. Autoimmun..

[78] Fujita H., Shemer A., Suarez-Farinas M., Johnson-Huang L.M., Tintle S., Cardinale I. (2011). Lesional dendritic cells in patients with chronic atopic dermatitis and psoriasis exhibit parallel ability to activate T-cell subsets. J. Allergy Clin. Immunol..

[79] Boyman O., Hefti H.P., Conrad C., Nickoloff B.J., Suter M., Nestle F.O. (2004). Spontaneous development of psoriasis in a new animal model shows an essential role for resident T cells and tumor necrosis factor-alpha. J. Exp. Med..

[80] Palmer C.N., Irvine A.D., Terron-Kwiatkowski A., Zhao Y., Liao H., Lee S.P. (2006). Common loss-of-function variants of the epidermal barrier protein filaggrin are a major predisposing factor for atopic dermatitis. Nat. Genet..

[81] Kabashima K. (2013). New concept of the pathogenesis of atopic dermatitis: interplay among the barrier, allergy, and pruritus as a trinity. J. Dermatol. Sci..

[82] Soumelis V., Reche P.A., Kanzler H., Yuan W., Edward G., Homey B. (2002). Human epithelial cells trigger dendritic cell mediated allergic inflammation by producing TSLP. Nat. Immunol..

[83] Nograles K.E., Zaba L.C., Shemer A., Fuentes-Duculan J., Cardinale I., Kikuchi T. (2009). IL-22-producing T22 T cells account for upregulated IL-22 in atopic dermatitis despite reduced IL-17-producing TH17 T cells. J. Allergy Clin. Immunol..

[84] Duhen T., Geiger R., Jarrossay D., Lanzavecchia A., Sallusto F. (2009). Production of interleukin 22 but not interleukin 17 by a subset of human skin-homing memory T cells. Nat. Immunol..

[85] Novak N., Allam J.P., Hagemann T., Jenneck C., Laffer S., Valenta R. (2004). Characterization of FcepsilonRI-bearing CD123 blood dendritic cell antigen-2 plasmacytoid dendritic cells in atopic dermatitis. J. Allergy Clin. Immunol..

[86] Romani N., Brunner P.M., Stingl G. (2012). Changing views of the role of Langerhans cells. J Investig Dermatol.

[87] Penel-Sotirakis K., Simonazzi E., Péguet-Navarro J., Rozières A. (2012). Differential capacity of human skin dendritic cells to polarize CD4+ T cells into IL-17, IL-21 and IL-22 producing cells. PLoS One.

[88] Matthews K., Chung N.P., Klasse P.J., Romani N., Brunner P.M., Stingl G. (2012). Changing views of the role of Langerhans cells. J Investig Dermatol.

[89] Igyarto B.Z., Haley K., Ortner D., Bobr A., Gerami-Nejad M., Edelson B.T. (2011). Skin-resident murine dendritic cell subsets promote distinct and opposing antigen-specific T helper cell responses. Immunity.

[90] Sancho D., Joffre O.P., Keller A.M., Rogers N.C., Martinez D., Hernanz-Falcon P. (2009). Identification of a dendritic cell receptor that couples sensing of necrosis to immunity. Nature.

[91] Dudziak D., Kamphorst A.O., Heidkamp G.F., Buchholz V.R., Trumpfheller C., Yamazaki S. (2007). Differential antigen processing by dendritic cell subsets in vivo. Science.

[92] del Rio M.L., Bernhardt G., Rodriguez-Barbosa J.I., Forster R. (2010). Development and functional specialization of CD103 dendritic cells. Immunol. Rev..

[93] den Haan J.M., Bevan M.J. (2002). Constitutive versus activation-dependent cross-presentation of immune complexes by CD8(+) and CD8(−) dendritic cells in vivo. J Exp Med.

[94] Edelson B.T., KC W., Juang R., Kohyama M., Benoit L.A., Klekotka P.A. (2010). Peripheral CD103+ dendritic cells form a unified subset developmentally related to CD8alpha+ conventional dendritic cells. J Exp Med.

